# Differential Regulation of Genes by the Glucogenic Hormone Asprosin in Ovarian Cancer

**DOI:** 10.3390/jcm11195942

**Published:** 2022-10-08

**Authors:** Rachel Kerslake, Cristina Sisu, Suzana Panfilov, Marcia Hall, Nabeel Khan, Jeyarooban Jeyaneethi, Harpal Randeva, Ioannis Kyrou, Emmanouil Karteris

**Affiliations:** 1 Division of Biosciences, College of Health, Medicine and Life Sciences, Brunel University London, Uxbridge UB8 3PH, UK; 2 Mount Vernon Cancer Centre, Rickmansworth Road, Northwood HA6 2RN, UK; 3 Warwickshire Institute for the Study of Diabetes, Endocrinology and Metabolism (WISDEM), University Hospitals Coventry and Warwickshire NHS Trust, Coventry CV2 2DX, UK; 4 Warwick Medical School, University of Warwick, Coventry CV4 7AL, UK; 5 Centre for Sport, Exercise and Life Sciences, Research Institute for Health & Wellbeing, Coventry University, Coventry CV1 5FB, UK; 6 Aston Medical School, College of Health and Life Sciences, Aston University, Birmingham B4 7ET, UK; 7 Laboratory of Dietetics and Quality of Life, Department of Food Science and Human Nutrition, School of Food and Nutritional Sciences, Agricultural University of Athens, 11855 Athens, Greece

**Keywords:** asprosin, ovarian cancer, OvCa, high grade serous ovarian cancer, HGSC, OR4M1, TLR4, metabolism, RNA sequencing

## Abstract

Background: Ovarian cancer (OvCa) is one of the most lethal forms of gynaecological malignancy. Altered energy metabolism and increased aerobic glycolysis in OvCa are hallmarks that demand attention. The glucogenic hormone asprosin is often dysregulated in metabolic disorders such as insulin resistance, diabetes (type 2 and gestational), and preeclampsia. Despite association with metabolic disorders, its role in energy metabolism within the tumour microenvironment is yet to be explored. Here, we study the role of asprosin in OvCa using transcriptomics and expand on functional studies with clinical samples. Methods: RNA sequencing, functional gene enrichment analysis, Western blotting and ImageStream. Results: Following treatment with 100 nM of asprosin, the serous OvCa cell line, SKOV-3, displayed 160 and 173 gene regulatory changes, at 4 and 12 h respectively, when compared with control samples (*p* < 0.05 and Log2FC > 1). In addition to energy metabolism and glucose-related pathways, asprosin was shown to alter pathways associated with cell communication, TGF-β signalling, and cell proliferation. Moreover, asprosin was shown to induce phosphorylation of ERK1/2 in the same in vitro model. Using liquid biopsies, we also report for novel expression of asprosin’s predicted receptors OR4M1 and TLR4 in cancer-associated circulating cells; with significant reduction seen between pre-chemotherapy and end of first line chemotherapy, in addition to patients under maintenance with bevacizumab +/− olaparib for OR4M1. Conclusions: In relation to OvCa, asprosin appears to regulate numerous signalling pathways in-vitro. The prognostic potential of OR4M1 in liquid biopsies should also be explored further.

## 1. Introduction

Asprosin is an orexigenic hormone involved in the stimulation of appetite as well as the regulation of hepatic glucose. As a c-terminal cleavage product of profibrillin-1, encoded by the gene FBN1, this protein is processed via furin mediated proteolysis [1]. Fasting induces elevated plasma asprosin which stimulates the release of hepatic glucose within the circulation, through G-protein coupled receptor (GPCR) mediated activation [2]. Asprosin is as an adipokine with production primarily localised within white adipose tissue (WAT),and elevated expression in obesity [3,4]; however, emerging studies also implicate asprosin expression with a growing number of peripheral tissues. 

Since its discovery as a glucogenic hormone, asprosin has been implicated in metabolic disorders such as obesity, insulin resistance (IR) and type 2 diabetes mellitus (T2DM) [5,6]. Moreover, asprosin is shown to express a sexually dimorphic profile, with case studies recording higher levels of plasma asprosin in females and showing fluctuations of plasma levels in association with anaerobic exercise for women, yet stable levels in men regardless of exercise [7]. 

Of note, female metabolic disorders appear to correlate frequently with asprosin dysregulation. Fluctuations of plasma asprosin are seen throughout phases of the menstrual cycle with lower levels reported in women taking progesterone-based oral contraceptives [8]. Asprosin is also associated with pathological pregnancy-related disorders such as preeclampsia and gestational diabetes [9,10]. The role of asprosin has also been studied in relationship to the pathophysiology of polycystic ovarian syndrome (PCOS) [11,12]. 

The expression profiling of asprosin is incomplete; to date, its expression outside WAT has been shown in normal ovarian stromal and epithelial tissues of heifers and mice [13,14]. Recently, we studied the expression of asprosin and its precursor gene, FBN1, in ovarian cancers (OvCa) of varying grade and subtype; including high grade serous OvCa, which accounts for approximately 80% of OvCa cases [15]. We expanded on these observations in normal human ovarian epithelial and OvCa cell lines as well as normal adjacent tissues (NAT). OvCa affects over 313,000 women globally [16]. Given that >70% patients with OvCa present too late for curative treatment, i.e., Stages III/IV, and that incidence is predicted to increase, efforts to understand the causes including the metabolic drivers of OvCa are vital [17,18].

Aerobic glycolysis, known as the Warburg effect, is an illustrious factor implicated in the progression of many cancers including OvCa [19]. Despite the presence of oxygen, preferential use of the glycolytic pathway, where glucose is used in the rapid production of energy, generating excess lactate, in opposition to aerobic respiration/oxidative phosphorylation, is often favoured utilising excessive glucose [20]. It is well recognized that respiration alone can maintain tumour viability. Aerobic glycolysis is a controllable factor, and aberrant regulation of growth factor signalling is an initiating event in oncogenesis [21]. As a glucogenic hormone, asprosin may prove to have a role in this process and is a promising candidate for investigation within the tumour microenvironment. 

Risk factors for OvCa include insulin resistance, diabetes, and obesity [17], factors aligned with the aforementioned disorders associated with dysregulated levels of asprosin. There are reports of asprosin in association with other malignancies, for example, basal cell carcinoma, pancreatic cancer, as well as ductal breast carcinoma [22,23,24], in addition to our earlier study in OvCa [15]. As such, asprosin provides a promising new candidate for exploration within the OvCa tumour microenvironment, as well as a potential therapeutic target. 

In the present study, we investigate changes at transcriptomic level (using RNAseq), in human OvCa cells treated with asprosin in vitro. This research builds upon previous work completed by our group, where we mapped the expression of asprosin within OvCa and presented evidence of the expression of a possible receptor of asprosin within OvCa using clinical samples and cell models [15]. Here, we aim to further elucidate the signalling pathways associated with asprosin using functional enrichment analyses of identified differentially regulated genes (DEGs) and explore its role within the tumour microenvironment.

## 2. Materials and Methods

### 2.1. Blood Samples and ImageStream Mark II Analysis

Blood samples from *n* = 100 OvCa patients were collected from Mount Vernon Cancer Centre, East and North Herts NHS Trust, as part of the CICATRIx study: Sample collection study to explore circulating tumour cells, cell free DNA and leucocytes with ImageStream analysis in patients with various cancers. The study was approved by the West Midlands–South Birmingham Ethics Committee (reference 16/WM/0196; protocol number RD2016-08). Samples were prepared for imaging as previously described, with the only difference being the substitution of Pan-Cytokeratin (AE1/AE3) antibody with that of OR4M1 or TLR4 [25]. Cell images were captured with the ImageStream^®^ X Mk II and subsequently assessed for the presence of OR4M1 or TLR4 using IDEAS^TM^ software.

Inclusion criteria: Female patients aged 18 y or more, histologically confirmed diagnosis of high-grade serous ovarian cancer (HGSC), availability of formalin-fixed, paraffin-embedded (FFPE) tissue taken at the time of diagnosis of cancer, able to comply with study procedures, life expectancy > 3 months, no contra-indications to blood sampling, biopsy, imaging, etc., and patients willing to anonymously share data and able to provide written informed consent.

Exclusion criteria: Patients with any other form of ovarian cancer, e.g., endometrioid, clear cell, mucinous carcinoma, borderline tumours; lack of written consent; or positive pregnancy test. OvCa staging is defined by the International Federation of Gynecology and Obstetrics (FIGO) staging system. Stage I cancer is confined to the ovaries. Stage II OvCa has metastasized to adjacent locations within the pelvic cavity. Stage III refers to OvCa metastasis outside of the pelvic cavity, and stage IV refers to distant metastases [26].

### 2.2. Tissue Culture

The SKOV-3 cell line (ECAAC 91091004) was grown in Dulbecco’s modified Eagle’s medium (DMEM), supplemented with 10% foetal bovine serum and 1% penicillin-streptomycin (Thermo Fisher Scientific, Loughborough, UK). Cells were grown in T75 filter head flasks at 37 °C, in humified conditions at 5% CO_2_ and passaged three times at 80–90% confluency before seeding in 6 well plates at a density of 0.3 × 10^6^. Cell count and viability assay were performed using a Neubauer chamber and Trypan blue (Invitrogen; Thermo Fisher Scientific, Loughborough, UK) exclusion method. Cells were treated with 100 nM of asprosin (Biolegend, San Diego, CA, USA) for 4 and 12 h in triplicate with corresponding controls. Duplicate experiments were generated for validation work. 

### 2.3. RNA Extraction and cDNA Synthesis

Treatments were arrested at 4 and 12 h following media removal and washed with PBS (Thermo Fisher Scientific, Loughborough, UK). RNA isolation was achieved using Qiagen RNeasy extraction kit (Qiagen, Manchester, UK), according to the manufacturer’s instructions; samples were eluted in 40 ul of deionised water. Purity assessment was performed with NanoDrop 2000C. RNA was stored at −80 °C prior to shipment and sequencing. Duplicate samples were reverse transcribed using Applied Biosystems High-capacity cDNA Reverse Transcription Kit (Thermo Fisher Scientific, Loughborough, UK), for RT-qPCR. 

### 2.4. RT-qPCR Analysis 

Relative gene expression of treated vs. control samples for 4 and 12 h were measured using iTaq^TM^ Universal SYBR Green Supermix (Bio-Rad Laboratories, Hercules, CA, USA) with the Bio-Rad CFX96 Touch Real-Time PCR Detection System, according to the provided guidelines. Primers used are listed in Table 1. Primer pairs for TXK were designed through Sigma Aldrich Primer Design with sequences obtained from the Harvard Primer Bank (Primer bank ID-148596973c3) [27]. Primers for MAGI2-AS3, FCGR2A and GAPDH were obtained commercially through Thermo Fisher Scientific. 

To quantify expression Ct values were used to calculate the expression fold change conveying the fold change in comparison to a valid calibrator, i.e., control samples. The ΔCt method was applied using the following formulae:

ΔCt = Ct gene of interest − Ct reference gene

ΔΔCt = ΔCt sample − ΔCt control average

Fold Change Expression = 2^−ΔΔCt^

### 2.5. RNA Sequencing 

Three technical replicates from each cohort (i.e., 3 × no supplement control and 3 × treated) were sent on dry ice for sequencing to Macrogen Seoul, South Korea. Samples underwent a strict quality control assessment (see appendix) before processing and cDNA library construction. Indexed libraries were submitted to an Illumina NovaSeq (Illumina, Inc., San Diego, CA, USA), and the paired-end (2 × 100 bp) sequencing was performed by Macrogen Incorporated using an Illumuna platform. Files were compiled by Macrogen using lllumina package bcl2fastq to convert the base call (BCL) binary results to FASTQ. The average reads of the triplicates for each condition are outlined in Table 2. 

An RNA seq processing pipeline was designed according to a previous study [28]. Briefly, TopHat2 (v.2.1.1) was used to align reads to the human reference genome, GRCH38 (hg19) with Bowtie2 (v.2.2.6) ultra-high-throughput short read aligner. All experimental replicates were merged using Samtools (v.0.1.19) with selection criteria of high-quality mapped reads set to <30, before transcript assembly and quantification using Cufflinks (v.2.2.1). Cuffdiff (v.2.2.1) was then used to obtain differential expression profiles between time points. 

### 2.6. RNA Sequencing Statistical Analysis 

Data processing, modelling, cleaning, visualisation, and statistical analyses were conducted using R (v. 4.1.0, The R Foundation for statistical Computing, Vienna, Austria) with the R Studio desktop application (version 1.4.1717, RStudio, Boston, MA, USA). Calculations include the Pearson correlation coefficient for estimation of gene correlation and expression pattern; student’s *t*-test to assess statistical significance between state of expression (e.g., asprosin 100 nM at 4 h vs. no supplement control at 4 h). Threshold for significance was set at a *p*-value < 0.05. Volcano plots and Venn diagram were generated using R package ggplot2 v.3.3.5. R package pathfindR was used for comprehensive identification of enriched pathways in omics data.

### 2.7. Functional Annotation 

Differentially expressed genes were characterised with FunRich (v.3.1.3), a software used for gene functional classification and annotation [29]. The following characteristics were computed for the associated DEGs: biological pathway, molecular function, biological process, cellular component, and site of expression. We also performed Gene Set Enrichment Analysis (GSEA), comparing controls vs. combined 4 and 12 h treatments, using the GSEA software [30].

### 2.8. Western Blot 

Protein lysate was extracted from treated cells using Laemmli buffer (Sigma Aldrich, Burlington, MA, USA). Samples were separated and transferred using SDS-PAGE gel electrophoresis and a wet transfer process. Membranes were blocked with 5% milk when detecting total proteins and 5% BSA when probing for phosphorylated proteins. Antibodies listed in Table 3 were left to incubate overnight at 4 °C. After washing with TBS Tween 20 (Sigma Aldrich, USA), the secondary antibody in a dilution of 1:2000 was applied for 60 min. Following additional washes, the proteins were exposed to X-ray film using enhanced chemiluminescence (Thermo Fisher Scientific, Loughborough, UK) and developed with Champion RG Universal RTU Developer and Champion RG Universal RTU Fixer solutions using an Agfa Curix 60 machine. Quantification was performed using Image J [31].

### 2.9. Immunohistochemical Staining 

An ovarian carcinoma tissue microarray containing 100 unique patient biopsy cores and adjacent tissues was purchased from Biomax Inc., Rockville, MD, USA (BC11115d). The ovarian tissue array contained 90 OvCa cores along with 10 non-adjacent tissue samples; it was not possible to obtain normal ovarian samples due to limited availability commercially, and ethical constraints obtaining extra healthy control tissues. Samples were probed with a 1:50 dilution of TLR4 antibody (Thermo Fisher Scientific, Loughborough, UK) according to our previously published methods [32]. All reagents unless otherwise stated were purchased from Thermo Fisher Scientific (Loughborough, UK). Briefly the FFPE slide was deparaffinized and rehydrated via submersion in histoclear and ethanol, followed by antigen retrieval in sodium citrate at 90 °C. Tissues were blocked with 5% BSA for 60 min (room temperature), before commencing overnight incubation at 4 °C with primary TLR4 antibody. The following day the slide was subjected to a series of washes with PBS 0.025% Triton X-100 prior to secondary antibody incubation (1:200 in 1% rabbit serum; ZytoChem Plus HRP-DAB kit, rabbit, cat. no. HRP008DAB-RB, Zytomed Systems GmbH) for 60 min (room temperature). Next, the slide was treated with streptavidin-HRP conjugate from the same Zytochem kit for 30 min. Final washes were conducted and the tissues were stained with 3,3′-diaminobenzidine (DAB) (Vector Laboratories, Inc., Newark, CA, USA) for 5 min and counterstained with haematoxylin (Merck KGaA, Darmstadt, Germany) for ~10 s before bluing with 0.1% sodium bicarbonate. Immunoreactivity of TLR4 was assessed using a light microscope (Carl Zeiss AG, Oberkochen, Germany). A pheochromocytoma (adrenal gland) core was used as a positive control.

## 3. Results

### 3.1. Differentially Expressed Genes (DEGs) upon Treatment with Asprosin

**Figure 1:**
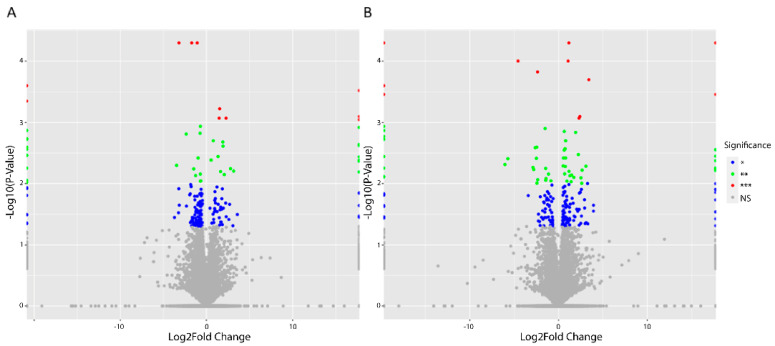
Volcano plots showing differentially regulated genes (DEGs) in SKOV-3 cells treated with 100 nM of asprosin. (**A**) DEGs 4 h post treatment; (**B**) DEGs 12 h following treatment. Significance level is recorded as NS (Non-Significant) = grey; * *p* < 0.05 = blue; ** *p* < 0.01 = green; *** *p* < 0.001 = red.

Following treatment with 100 nM of asprosin, the serous OvCa cell line, SKOV-3, displayed 160 and 173 gene changes, at 4 and 12 h, respectively, when compared with control samples extracted at parallel time points, *p* < 0.05 and Log2FC > 1. DEGs were identified using the Cuffdiff multiple-testing module [28]. These changes are mapped in the volcano plots presented in Figure 1.

### 3.2. Functional Analysis of DEGs

**Figure 2:**
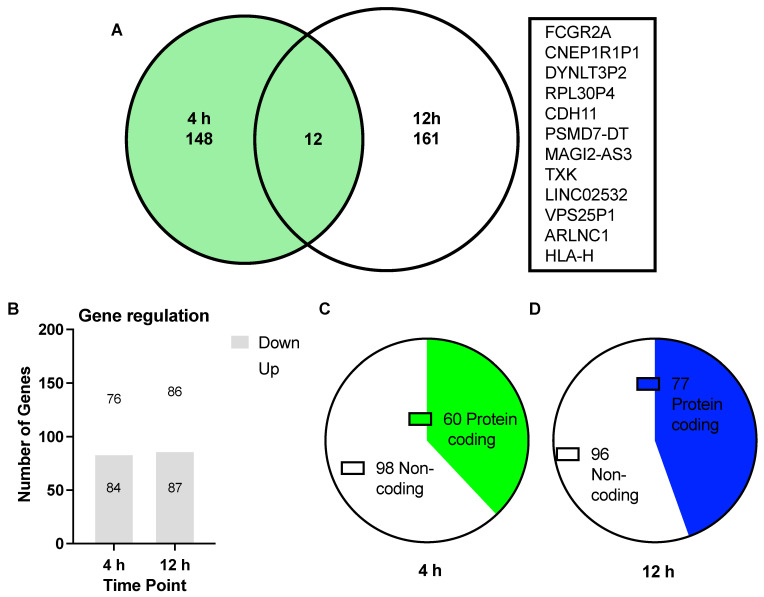
Summary of differentially expressed genes (DEGs) from 4 h (green) and 12 h (blue) data sets, comparing SKOV-3 cells treated with 100 nM of asprosin with non-supplemented controls. (**A**) Venn diagram depicting the number of unique DEGs at 4 h (148) and 12 h (161) with 12 common genes between the overlapping time points annotated to the right of the diagram; (**B**) Bar graph showing the number of up regulated and down regulated DEGs at each time point; (**C**,**D**) Quantification of coding and non-coding genes for each time point: (**C**) 60 protein coding, with 98 non-coding at 4 h; (**D**) 77 protein coding and 96 non-coding at 12 h.

A combination of 321 genes from the SKOV-3 cells were presented as being differentially regulated from the control groups when treated with 100 nM of asprosin compared with control samples extracted at parallel time points. A total of 160 of these DEGs were identified 4 h post treatment and an additional 173 DEGs were detected 12 h following treatment. All genes chosen for functional enrichment analysis were subjected to a threshold of *p* < 0.05 and Log2FC > 1. There were 12 common DEGs between the 4 h and 12 h groups (Figure 2). 

**Figure 3:**
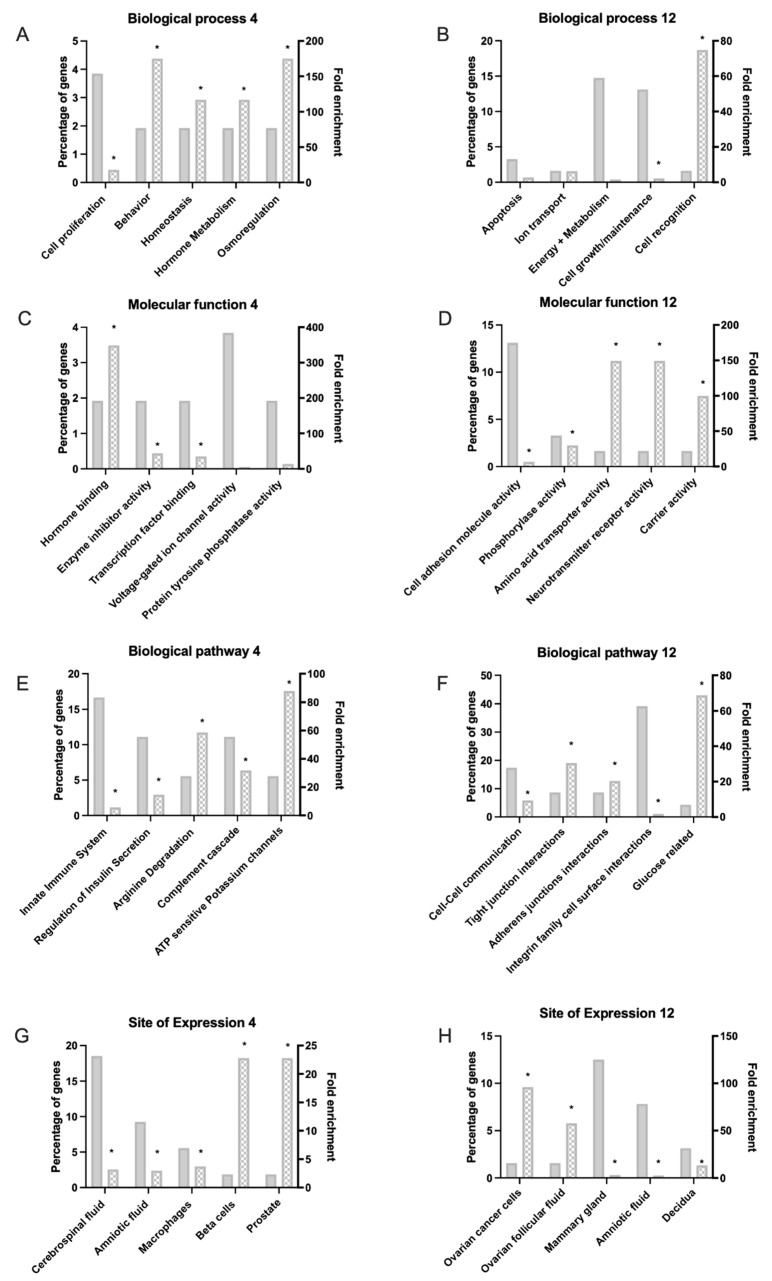
Functional enrichment of differentially expressed genes (DEGs) matched with Funrich data base. (**A**,**C**,**E**,**G**) indicate altered process 4 h after treatment with 100 nM asprosin; (**B**,**D**,**F**,**H**) show processes associated with DEGs 12 h after treatment. (**A**,**B**) Biological process; (**C**,**D**) Molecular function; (**E**,**F**) Biological Pathway; (**G**,**H**) Site of expression. Significant data sets identified using hypo geometric test are indicated by * *p* < 0.05.

Using the Funrich functional annotation data base (version 3.1.4), 58/160 and 64/173 DEGs were recognised for functional annotation from the 4 and 12 h data sets, respectively. Classification of the biological processes, molecular function, biological pathways, sites of expression and cellular components associated with asprosin treatment were identified with fold change expression measured and hypo geometric analysis applied, * *p* < 0.05 (Figure 3).

**Figure 4:**
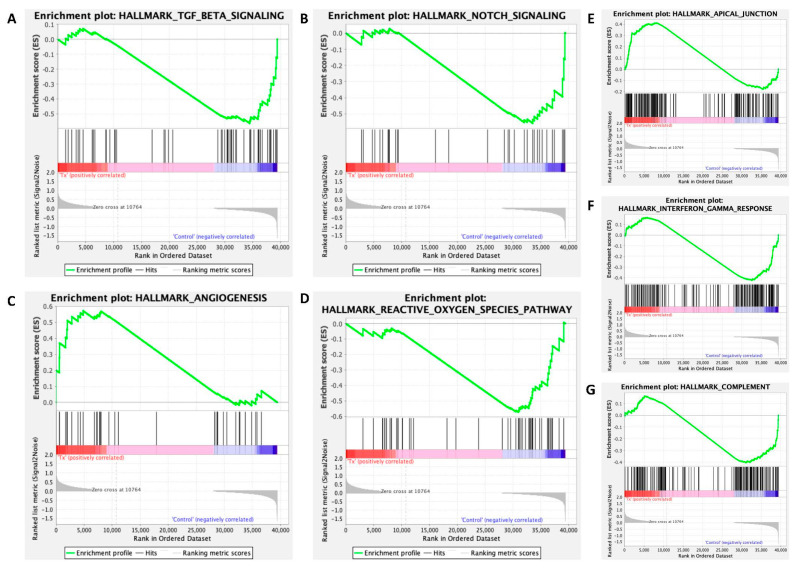
Gene set enrichment analysis (GSEA) of 100 nM asprosin-treated cells at 4 and 12 h combined panel of enrichment score curves: (**A**) TGF Beta Signalling; (**B**) Notch Signalling; (**C**) Angiogenesis; (**D**) Reactive Oxygen Species (ROS); (**E**), Apical Junction; (**F**) Interferon Gamma Response; (**G**) Complement.

**Figure 5:**
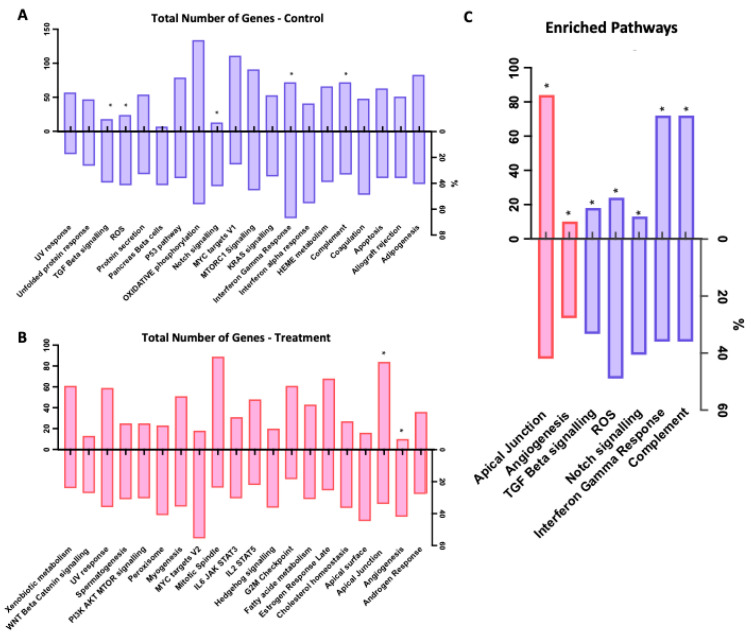
Selection of GSEA identified pathways influenced by asprosin in OvCa treated with 100 nM at 4 and 12 h combined. (**A**) Total pathways influenced by asprosin for control group (down regulated) in blue; (**B**) Total pathways influenced by asprosin for treatment group (up regulated) in red; (**C**) Combined pathways (regulation: up = red, down = blue) with pre-defined GSEA automated nominal *p* set to * *p* = 0.2.

GSEA analysis showed a number of pathways regulated by asprosin, including apical junction, angiogenesis, TGF-β and notch signalling, reactive oxygen species (ROS) and complement (Figure 4 and Figure 5). 

### 3.3. Validation of Differentially Expressed Genes

**Figure 6:**
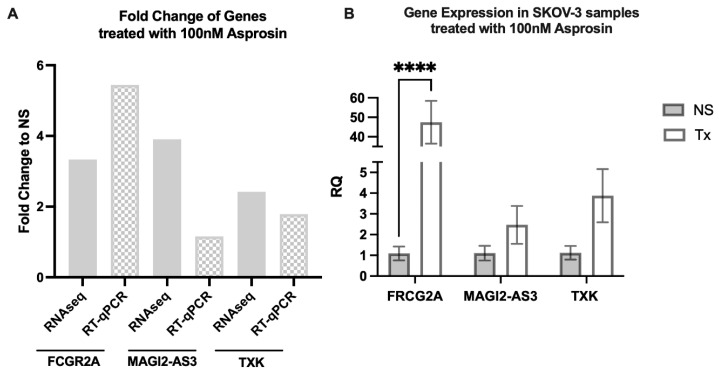
Validation of RNA seq data. Expression of FRCG2A, MAGI2-AS3 and TXK in SKOV-3 cells following treatment (Tx) with 100 nM asprosin at 4 h. (**A**) Gene expression shown as fold change (FC) in gene expression following treatment (100 nM asprosin) at 4 h compared to RNA seq fold increase. (**B**) RT-qPCR analysis of cells treated (Tx) with asprosin (100 nM) versus no supplement (NS) showing an increase in trend for all genes, with a significant change seen for FRCG2A, **** *p* < 0.0001.

Genes that were commonly expressed at both 4 and 12 h were chosen for analysis, in addition to their association with cancer in the literature [33,34,35]. FCGR2A, MAGI2-AS3 and TXK were up-regulated on initial treatment with asprosin and show a similar increase in expression upon repeat and validation with RT-qPCR, albeit to a lesser effect than seen in RNA sequencing (Figure 6). 

### 3.4. Asprosin Induces Phosphorylation of ERK1/2

**Figure 7:**
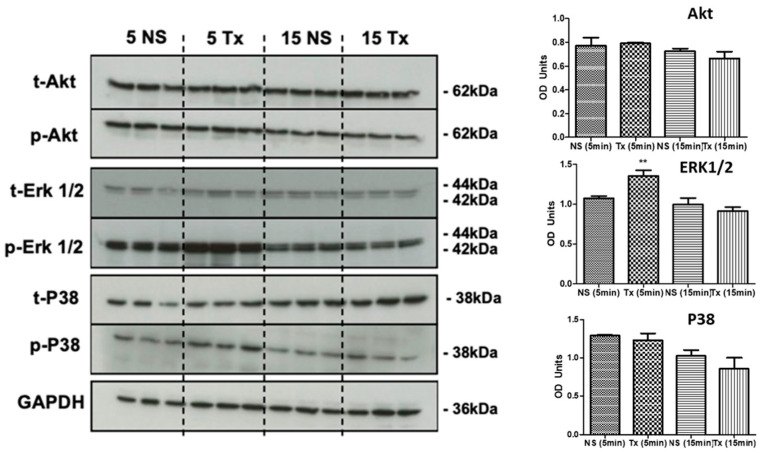
Western blots showing the total and phosphorylated proteins Akt, ERK 1/2, P38, and the loading control GAPDH following treatment with 100 nM of asprosin terminated at 5 and 15 min; followed by densitometric analysis. OD: Optical density, ** *p* < 0.01.

SKOV-3 cells were treated with asprosin (100 nM) for 5 and 15 min and phosphorylation of key kinases was measured using Western blotting. With the exception of ERK1/2, all other kinases (Akt, and p38) did not show an increase in phosphorylation within the times assessed (Figure 7). The increase in ERK1/2 phosphorylation appeared to be significant at 5 min.

### 3.5. Cancer-Associated Circulating Cells Express Receptors for Asprosin

**Figure 8:**
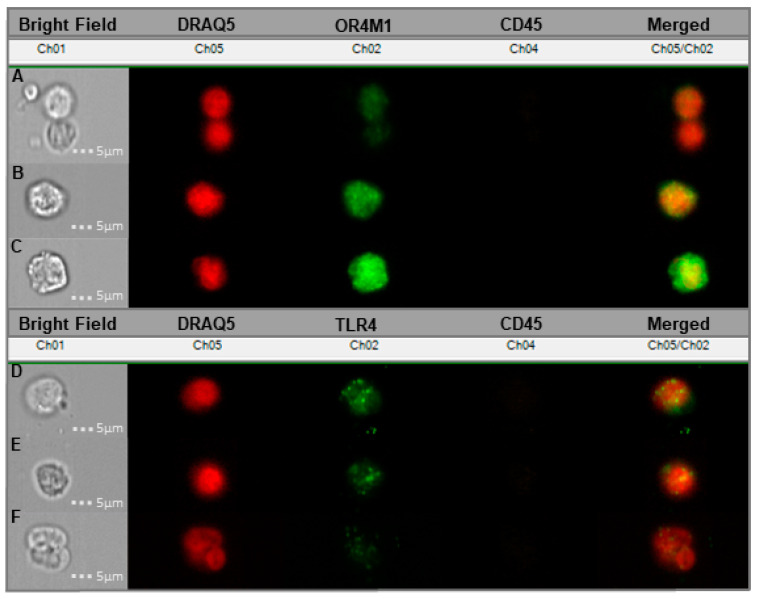
Cancer-associated circulating cells (CCs) from liquid blood biopsies donated by six different serous OvCa patients probed with OR4M1/TLR4. (**A**–**C**) Samples showing the expression of OR4M1 (Ch02—green) in the circulating tumour cells of patients presenting with high grade serous ovarian cancer; (**A**) represents stage IV, (**B**) stage IV and (**C**) stage III; (**D**–**F**) Expression of TLR4 in CCs; here, Ch02 represents TLR4 (green); (**D**) is representative of stage II, (**E**) stage III and (**F)** stage IV. Ch01, Bright Field; Ch02, OR4M1/TLR4 (green); Ch04, CD45—white blood cell exclusion marker (brown); Ch05, DRAQ5—cell nuclear stain (red); Ch02/05, overlay of Ch02 with Ch05. Stages (I–IV) refer to the severity of OvCa metastasis according to the International Federation of Gynaecology and Obstetrics (FIGO) staging system.

In our previous study [15], we demonstrated that OvCa tissue expresses OR4M1. Here, we expand on these initial observations, showing that OvCa associated circulating cells express both of the considered receptors for asprosin, OR4M1 and TLR4 (Figure 8). 

**Figure 9:**
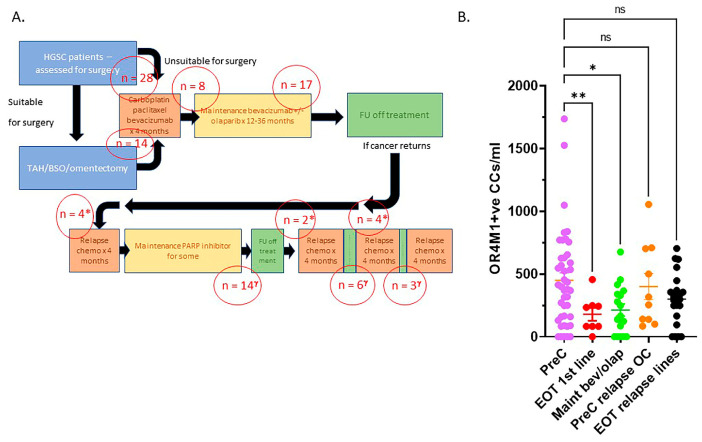
Panel (**A**): Schema of the recruitment of patients. FU: follow up, TAH/BSO: abdominal hysterectomy and bilateral salpingo-oophorectomy. Panel (**B**): OR4M1 + ve circulating cells decrease with chemotherapeutic treatment; 100 patients, representative of patients presenting with high grade serous ovarian cancer (HGSC) stages: I–IV: Pre-chemo (PreC, *n* = 42), end of first line chemotherapy (EOT 1st line, *n* = 8), maintenance bevacizumab +/− olaparib (maint bev/olap, *n* = 17), pre-chemo for relapse OC (PreC relapse OC, *n* = 10), end of chemo for relapse OC (EOT relapse lines, *n* = 23). (Error bars: SEM; significance determined using ANOVA, * *p* = 0.012, ** *p* = 0.0069). CCs: cancer-associated circulating cells.

Moreover, in the case of OR4M1 + ve circulating cells, there is a significant downregulation as treatment progresses. In this study, 100 patients with high grade serous OvCa, being managed according to standard UK practise, had blood samples taken at various points during their cancer treatment (Figure 9A). The median age was 70 years (range 35–87). All except 7 patients were diagnosed with Stage III or IV high grade serous OvCA. Twenty-eight patients had samples taken at diagnosis prior to starting any treatment at all (pre chemotherapy or PreC). Fourteen patients had samples taken after their primary surgery and prior to any adjuvant chemotherapy treatment. We have grouped these patients together as the pre-chemotherapy cohort (*n* = 42). 

Eight patients had samples taken following completion of their first line chemotherapy treatment (carboplatin/paclitaxel +/− bevacizumab) and any surgery but prior to starting their maintenance bevacizumab (end-of-treatment first line cohort or EOT 1st line). Another 17 patients had samples taken during the maintenance treatment following first line chemotherapy and any surgery (maintenance bevacizumab/+/−olaparib or maintenance bevacizumab /olaparib).

Ten patients (*) had samples taken prior to commencing chemotherapy treatment for relapse HGSC (PreC for relapse). Four had blood samples taken prior to treatment for first relapse (second line chemotherapy), 2 prior to treatment for second relapse (third line of chemotherapy) and four patients prior to fourth/fifth line of chemotherapy treatment. Finally, 23 patients (γ) had blood taken at the end of chemotherapy treatment for a relapse of their cancer (EOT relapse lines): 14 following treatment for first relapse, most of these were on maintenance PARP inhibitors, 6 following third line chemotherapy (second relapse) and 3 following fourth/fifth line chemotherapy for third/fourth relapse.

Figure 9B demonstrates the numbers of OR4M1 positive CCs/mL per category. There was an average of 450 ± 60 CCs for the pre-chemotherapy cohort, 179 ± 51 for the end of first line chemotherapy cohort, 213 ± 48 for maintenance bevacizumab/+/− olaparib cohort, 400 ± 104 for PreC relapse OvCa and 300 ± 43 for EOT-relapse lines. There were no differences in the expression OR4M1 positive CCs in BRCA wt (*n* = 90, 324 ± 28) versus BRCAm (*n* = 10, 419 ± 117) patients’ samples.

## 4. Discussion

Despite a growing association with metabolic disorders, few forays have yet been made to explore asprosin’s role in cancers. Our present work builds upon our previous findings of asprosin expression in OvCa tissue and starts to elucidate the role, if any, that asprosin may elicit over the Warburg effect and the tumour microenvironment [15]. In this study, we provide novel evidence on how asprosin can affect the OvCa transcriptome in vitro. In total, 160 and 173 differentially expressed genes (DEGs) were identified following exposure of SKOV-3 cells to 100 nM of asprosin at 4 and 12 h, respectively. Only 12 of these DEGs were similarly expressed at both time points. These DEGs include: the functionally expressed FCGR2A, CDH11 and TXK; the non-coding RNAs PSMD7-DT, MAGI2-AS3, LINC02532 and ARLNC1; as well as pseudogenes CNEP1R1P1, DYNLT3P2, RPL30P4, VPS25P1 and HLA-H. 

Of the functionally expressed genes, many present associations with cancers throughout the literature. FCGR2A, for example, a gene involved in immune response and phagocytosis, is associated with chemotherapeutic and disease response in OvCa [33]. While increased expression, along with diminished CA125, also correlates positively with the survival of patients undergoing relapse, through the provision of enhanced farletuzumab receptor affinity [36]. Increased regulation of FCGR2A in our data following treatment with asprosin (Figure 2), may present a role in therapeutic response that requires future exploration. Furthermore, Cadherin 11 (CDH11), a membrane protein involved in calcium-dependant cell adhesion, essential for bone development and maintenance, is capable of regulating proliferation via ERK 1/2 signalling pathways [37,38]. In OvCa, CDH11 is connected to advanced stage and nodal involvement [39], in addition to migration and metastasis [37], yet displays limited involvement in cancer progression. CDH11 in association with lactic acid, a key metabolite of the Warburg effect, is, however, implicated in the metastatic progression of colorectal cancer [40]. Our research shows an increase in CDH11 expression within OvCa when exposed to asprosin, perhaps indicating association with the Warburg effect. Additional investigation with Kaplan Meier (Km) plotter links an increase of this DEG with poor overall survival (OS) in OvCa, *p* < 0.002 (Supplementary Figure S1). In addition, Yang et al. show that increased expression influences paclitaxel resistance in gastric cancer [41]. The tyrosine kinase, TXK, a signalling molecule involved in T Helper 1 cytokine production, is a predicted inhibitor of proliferation in cancers such as breast and colon [35,42,43]. Asprosin elicits an increase of this gene in our data, perhaps regulating cell growth, while Kaplan Meier analysis correlates high expression positively with OS (*p* < 1.5 × 10^−6^).

Non-coding RNAs are increasingly thought to be integral for gene regulation and are emerging targets of cancer biology. For example, the DEG, LINC02532, was recently identified as a marker of radiosensitivity in clear cell renal carcinoma, while ARLNC1, decreased in our data, is associated with maintenance of androgen receptor signalling in prostate cancer [44,45]. The ncRNA MAGI2-AS3 is ubiquitously expressed in many cancers including OvCa and is another regulator of cell proliferation [46]. Gokulnath et al. show that MAGI2-AS3 can act as a tumour suppressor in high-grade serous carcinoma (HGSC) through the sponging of microRNAs and the suppression of MYC, leading to an inhibition of cell proliferation and migration [34,47]. Asprosin is seen to increase the expression of this gene at both 4 and 12 h. Increased expression, however, is associated with attenuated response to therapeutics such as cisplatin in nasopharyngeal carcinoma as well as lapatinib [48]. Further exploration with a Km plotter also associates increased expression with poor OS *p* < 0.018 (Supplementary Figure S1), perhaps implying biomarker traits. Further research is needed to understand the complex role of this DEG in OvCa. 

Moreover, with the exception of HLA-H, which is associated with cervical carcinoma [49], the pseudogenes listed above reveal no hits within the literature (i.e., PubMed circa 20 June 2022). Of note, there were 194 non-coding genes identified following treatment with asprosin consisting of lncRNAs and pseudogenes. The functional classification of many pseudogenes remains to be determined, although there is literature suggesting that pseudogenes are often aberrantly expressed in cancers [50]. Indeed, our group has recently demonstrated a functional role of the lncRNA X-inactive specific transcript (XIST) in lung cancer [51]. Further investigation within the field is required to categorize these genes and the role they play in the ovarian tumour microenvironment. 

Functional enrichment presented classifications for 58 of the 160 DEGs from the 4 h data set and 64 of the 173 DEGs from the 12 h data set. Our analysis revealed asprosin mediated dysregulation of key biological processes and hallmarks of cancer such as: apoptosis, cell proliferation and growth, as well as energy and metabolism [52]. The tumour microenvironment is known to be modulated by oncogenes which in turn regulate certain aspects of metabolism, ultimately leading to uncontrolled proliferation and metastasis [53].

Molecular functions dysregulated by asprosin are presented in Figure 3C,D, and include hormone binding, cell adhesion as well as protein tyrosine phosphatase activity; a process recently associated with asprosin mediated appetite stimulation [54]. Additional processes detected indicate that asprosin related DEGs are connected to the regulation of insulin as well as glucose, which align with asprosin’s metabolic profile as a glucogenic hormone capable of influencing homeostasis, its role following OR4M1 binding, as well as asprosin’s role in insulin resistance [6]. Of note, elevated glucose levels along with increased expression of the glucose transporter, GLUT1, are potential biomarkers for OvCa [55]. The sites of expression with DEGs showing the highest degree of significance at 12 h are highly associated with female reproductive tissues including: OvCa; ovarian follicular fluid; mammary gland and decidua. This expression profile is to be expected of a hormone increasingly implicated with female reproductive disease and further emphasizes the need to assess asprosin’s importance in the female metabolic profile. 

Gene set enrichment analysis (GSEA) revealed asprosin mediated dysregulation of additional pathways: apical junctions, angiogenesis, transforming growth factor beta (TGF-β), notch signalling, reactive oxygen species (ROS), interferon gamma (IFN-γ) response and complement systems. 

Claudins such as CLDN9 and 18 maintain polarity in epithelial/endothelial cells and are integral for the formation of tight cellular junctions known as apical junctions (Figure 4E). These junctions are vital for the polarization of epithelial cells and effective cell communication [56]. CLDN9 and CLDN18 show core enrichment through GSEA analysis in our data sets, Log2FC = 2 and Log2FC = 1.5, respectively. CLDN9 is associated with aerobic glycolysis (Warburg effect) in gastric and endometrial cancers and is associated with poor prognosis in oesophageal cancer [57,58]. CLDN18 has recently been established as a normal gastric tissue marker; however, the presence of a splice variant has been documented in patients with mucinous ovarian cancers, a rare subtype with a poor outlook [59,60]. The over-expression of CLDN18.2 in intestinal type mucinous tissue acts as a potential biomarker of mucinous borderline ovarian tumours, distinguishing Mullerian types, where it is absent, from intestinal sub-types [59,61]. 

The GSEA-associated pathways, angiogenesis and ROS, are well documented as hallmarks of cancer; OvCa is particularly responsive to anti-angiogenic agents such as bevacizumab [62]. ROS is often dysregulated in cases of hypoxia promoting DNA damage and genomic instability, another key hallmark of high grade serous OvCa [63]. Dysregulation of the Notch pathway in OvCa is indicative of poorer prognosis and increased chemo-resistance [64,65]. 

TGF-β signalling increases cellular proliferation, epithelial to mesenchymal transition (EMT), and reduces apoptosis. In OvCa, TGF-β regulates cell proliferation through activation of the IGF1R signalling axis, with increased levels associated with poor survival outcome [66]. TGF-β signalling is regulated by Fibrillin-1, the primary product of asprosin’s precursor protein profibrillin-1 [67]. Aberration of TGF-β signalling is associated with Marfan’s syndrome, an autosomal dominantly inherited connective tissue disorder categorized by skeletal, ocular, and cardiovascular anomalies. Marfan’s is associated with mutations in the Fibrillin-1 gene, FBN1; other phenotypic variants such as marfanoid progeroid lipodystrophy syndrome, and neonatal progeroid syndrome form part of this group of ‘fibrinillopathies’ and are associated with depletion of asprosin, consequently encoded by the final two exons of FBN1 [68,69]. As such, dysregulation of this pathway may indicate an asprosin exerted feedback mechanism in OvCa.

To evaluate the effects of asprosin on common signalling pathways identified in the functional enrichment analysis we explored the phosphorylation status of Akt, ERK 1/2 and P38 using Western blot analysis. Following treatment with 100 nM asprosin, the SKOV-3 model did not demonstrate any change in Akt phosphorylation, 5 or 15 min after asprosin was administered. Nor were there marked changes for p38, an integral molecule involved in cell death which is associated with female cancers [70]. Our data does, however, indicate a short-lived increase in the phosphorylation of ERK 1/2, 5 min after asprosin treatment (Figure 7). The aforementioned, CDH11, which is increased in our data, is an emerging regulator of ERK 1/2. Heightened phosphorylation of ERK 1/2 is associated with increased cell proliferation in cancers including ovarian [71]. This is the first time that changes in ERK 1/2 phosphorylation have been associated with asprosin treatment; as such, its role as a downstream target requires further attention. Despite our study recording no change in Akt phosphorylation for the measured time scales, there are reports of impaired phosphorylation, along with insulin resistance, in mouse skeletal muscle treated with 100 nM of asprosin 24 h after treatment. Therefore, additional longer time points may reveal further post-translational modifications in addition to our short-lived burst of ERK 1/2 phosphorylation; as such, these signalling molecules should not be discounted from future research. 

Asprosin appears through the literature to bind promiscuously to three diverse receptors, each responsible for a distinct biological response. Indeed, binding to Olfactory Receptor 4 Member 1 (OR4M1), a G-protein coupled receptor (GPCR), implicates asprosin with glucose regulation; binding to Toll Like Receptor 4 (TLR4), which is aberrantly expressed in OvCa tissues of varying stages, grades and subtypes (Supplementary Figure S2), associates asprosin with insulin resistance in skeletal muscle [2,72]; emerging evidence also implicates stimulation of Protein Tyrosine Phosphatase Receptor delta (PTPRd) by asprosin as a mediator of orexigenic influence [54]. Additional work should seek the use of cross-linked proteins with immunoprecipitation and mass spectrometry to identify which, if not all, receptors asprosin binds to in a cell- or organ-specific manner. However, this falls beyond the scope of the current study as first we sought to explore the expression profile of these receptors. 

Here, using liquid biopsies from OvCa patients, we demonstrate novel expression of both OR4M1 and TLR4 in cancer-associated circulating cells (CCs) of patients with high grade serous OvCa, with a significant decline in OR4M1 positive cells seen between pre-chemotherapy and treatments. To the best of our knowledge, this is the first time an olfactory receptor demonstrates a prognostic potential in OvCa. Due to limitations of samples, we could not replicate the study for TLR4, and we acknowledge this limitation. Interestingly, TLR4 expression has been linked with cellular proliferation and paclitaxel resistance in vitro [73]. The presence of both predicted receptors on the surface of these cells may indicate another potential role of this energy metabolite in cancer-associated circulating cells. 

Due to ethical restrictions, we were unable to compare levels of TLR4 and PTPRd. We acknowledge that this is a major limitation of this part of the study, and additional quantification of asprosin-associated receptors TLR4 and PTPRd in liquid biopsies and correlation with progression of treatment will encompass impending studies. Another limitation of the study is the use of a singular OvCa cell line. In the future, other OvCa preclinical models including HEYA8, or OVCAR5 or OVCAR8 should be used to investigate further the effects of asprosin. Future studies should concentrate on performing analysis using a wider repertoire of OvCa in vitro models focussing on metabolic pathways using glycolysis assay and lactate production; in addition, a Seahorse assay should be applied to see how asprosin modulates these functions under normal conditions and following silencing of OR4M1, TLR4 or PTPRd receptors using CRISPR or siRNA. Future studies should also concentrate on assessing changes in the glucose uptake rate and lactate production upon treatment with asprosin in vitro. Finally, further validation of the metabolic pathways from GSEA analyses using a number of in vitro models will be useful. In particular, genes that were highlighted under GSEA analyses and also appeared as significant DEGs such as: ABCC8, that have shown prognostic significance in OvCa, or CLDN18 or 9, given the role of claudins in tumorigenesis require future exploration.

## 5. Conclusions

This study presents asprosin as a hormone capable of influencing gene regulation within the ovarian tumour microenvironment. 160 and 173 genes were dysregulated following treatment with 100 nM of asprosin in the OvCa cell model SKOV-3, at 4 and 12 h, respectively. Enrichment analysis revealed dysregulated pathways associated with energy metabolism such as oxidative phosphorylation, glucose regulation, ATP channels, glycolysis as well as ROS, in addition to genes such as FCGR2A, CDH11, MAGI2-AS3 as well as CLDN9 and 18. Our annotation accentuates asprosin’s role in energy metabolism and presents evidence of possible influence over genes associated with the Warburg effect within the ovarian tumour microenvironment as well as asprosin’s potential for further exploration in relation to therapeutic response. The mediation of these pathways by asprosin needs to be explored further to produce a definitive mechanism of action. Our research highlights the importance of asprosin as an emerging regulator of the female-specific metabolic profile. 

## Figures and Tables

**Table 1:** Primer sequences used for RNA seq validation.

Gene	Strand F to R
TXK	ACGGAGGCTGCCATAAAACAT
	GGATTGATTGAAAGGCGTGTCT
FCGR2A	CCTGAGAGCGACTCCATTCAG
	GTCTGTAAACAGATTTCATCCGTCCT
MAGI2-AS3	GCTCTCATAGGCCACCTTGC
	CTCCATCCTCATTCTCTCACCAC
GAPDH	GGAGAAGGCTGGGGC
	GATGGCATGGACTGTGG

**Table 2:** Total number of reads. For paired-end sequencing values refer to the average sum of read 1 and read 2 in triplicate.

Condition	Average Total Reads
Control (4 h)	70,027,291
100 nM Asprosin (4 h)	69,128,541
Control (12 h)	68,811,451
100 nM Asprosin (12 h)	74,371,331

**Table 3:** List of antibodies used in Western blot.

Antibodies	Dilution	Source
GAPDH	1:1000	Cell Signalling
ERK 1/2	1:1000	Thermo Fisher Scientific
P38	1:1000	Cell Signalling
Akt	1:1000	Cell Signalling
Phospho-ERK 1/2	1:1000	Thermo Fisher Scientific
Phospho-P38	1:1000	Cell Signalling
Phospho-Akt	1:1000	Cell Signalling

## Data Availability

RNAseq data available upon reasonable request.

## Supplementary Materials

The following supporting information can be downloaded at: https://www.mdpi.com/article/10.3390/jcm11195942/s1, Figure S1: Kaplan Meier plots showing overall survival (OS) of OvCa patients with dysregulation of the following genes; Figure S2: Immunohistochemical Staining for TLR4 in ovarian cancers and control tissues; Figure S3. Raw data from Western blotting experiments. (**A**), Total and phospho-Akt; (**B**), Total and phos-pho-p38; (**C**), Endo Total and phospho-ERK1/2; (**D**), GAPDH as loading control.
