# Supplementary material for: Differential Regulation of Genes by the Glucogenic Hormone Asprosin in Ovarian Cancer
Source: J Clin Med. 2022 Oct 8;11(19):5942. doi: 10.3390/jcm11195942 (PMC9573256; doi:10.3390/jcm11195942)
Supplement: Supplementary file 1 [file jcm-11-05942-s001.zip › jcm-1876203-supplementary.pdf]

Journal of
Clinical Medicine

MDPI

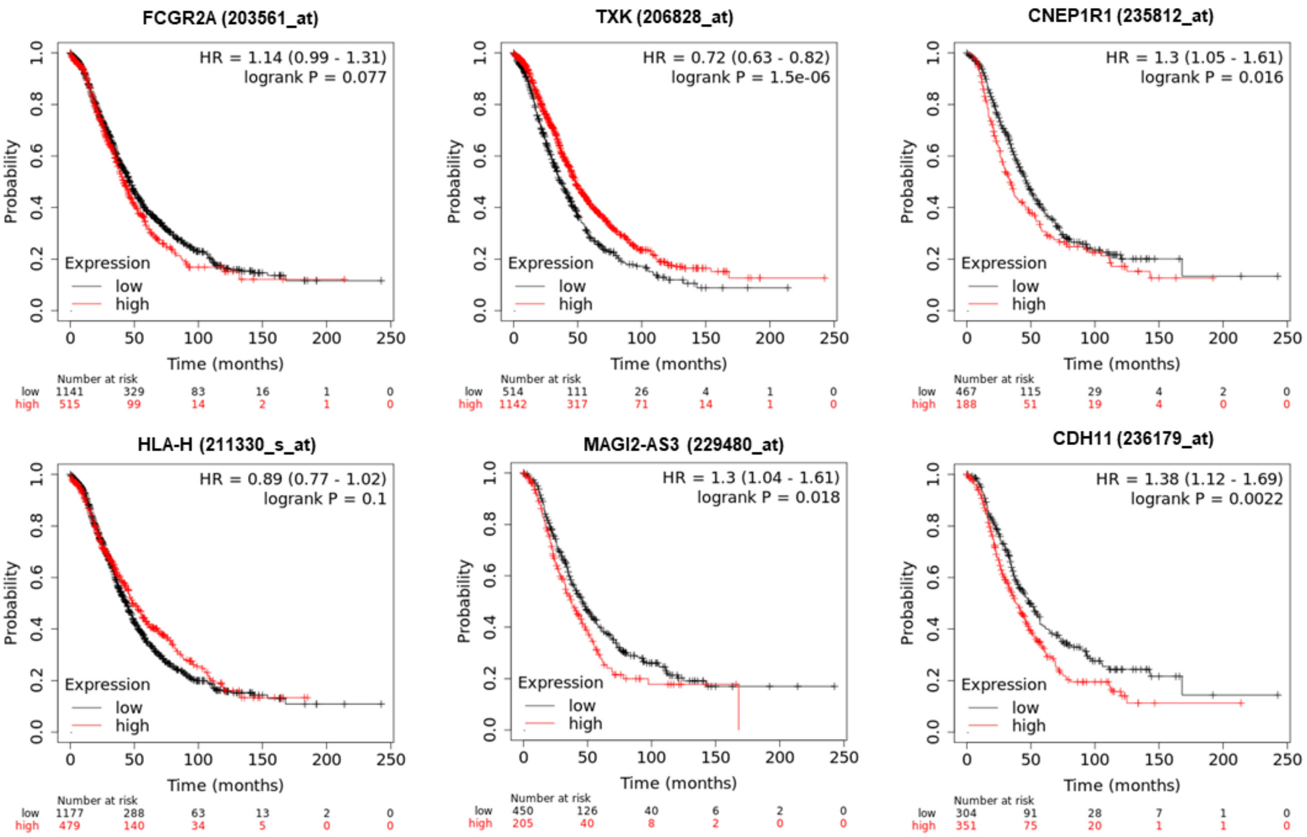

Supplementary Figure S1. Kaplan Meier plots showing overall survival (OS) of OvCa patients with dysregulation of the following genes: A, FCGR2A (p = not significant); B, TXK (*p = 000001.5); C, CNEP1R1 (*p = 0.016); D, HLA-H (p = not significant); E, MAGIC-AS3 (*p = 0.018); F, Alb (*p = 0.0022).

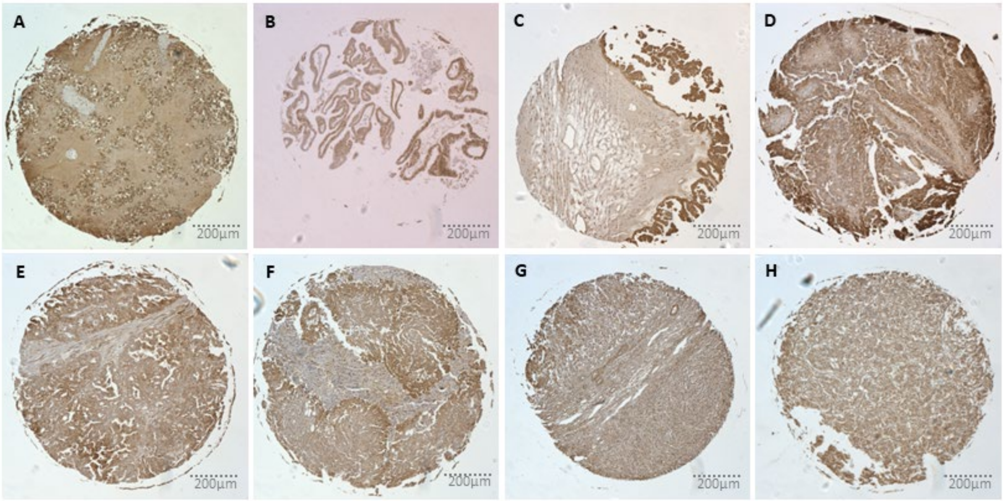

Supplementary Figure S2. Immunohistochemical Staining for TLR4 in ovarian cancers and control tissues at x10 magnification. **A**. Clear Cell Carcinoma, stage II; **B**, Low Grade Serous Carcinoma, stage II; **C**, Endometroid adenocarcinoma, stage I; **D**, High Grade Serous Carcinoma, stage III; **E**, Mucinous Adenocarcinoma, stage III; **F**, Lymph node metastasis from Ovary; **G**, Normal adjacent ovarian tissue; **H,** Control tissue Adrenal Pheochromocytoma.

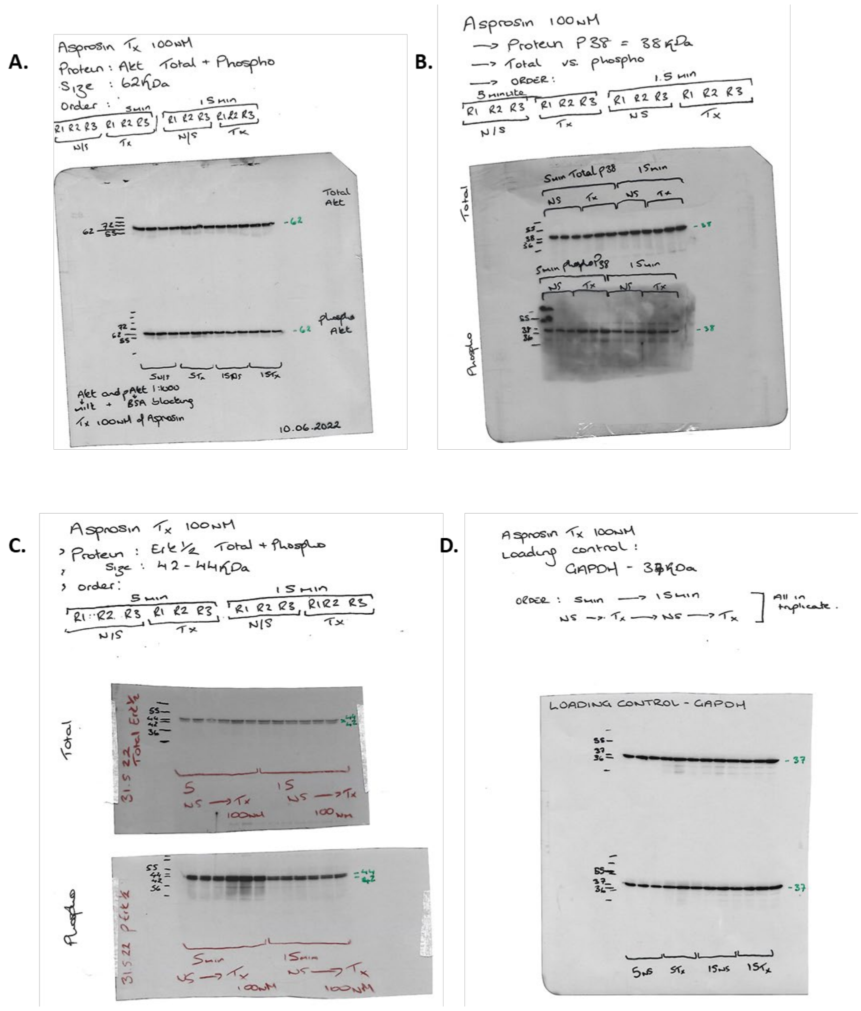

Supplementary Figure S3. Raw data from Western blotting experiments **A**. Total and phospho-Akt; **B**, Total and phospho-p38; **C**, Endo Total and phospho-ERK1/2 **D**, GAPDH as loading control.
